# 1-(4-Bromo­phen­yl)-3-butano­ylthio­urea

**DOI:** 10.1107/S1600536810050373

**Published:** 2010-12-08

**Authors:** Sohail Saeed, Naghmana Rashid, Jerry P. Jasinski, Ray J. Butcher, Muhammad Shoaib

**Affiliations:** a Department of Chemistry, Research Complex, Allama Iqbal Open University, Islamabad, Pakistan; b Department of Chemistry, Keene State College, 229 Main Street, Keene, NH 03435-2001, USA; c Department of Chemistry, Howard University, 525 College Street NW, Washington, DC 20059, USA; d National Engineering & Scientific Commission, PO Box 2801, Islamabad, Pakistan

## Abstract

In the title compound, C_11_H_13_BrN_2_OS, there are two independent mol­ecules (*A* and *B*) in the asymmetric unit. The dihedral angle between the mean planes of the benzene ring and the carbamothioyl group is 63.66 (mol­ecule *A*) and 80.3 (0)° (mol­ecule *B*). The butanamide group in mol­ecule *A* is disordered [0.532 (6) and 0.468 (6) occupancy]. The carbamothioyl group is twisted by 63.6 (6) (mol­ecule *A*) and 80.3 (0)° (mol­ecule *B*) from the respective benzene ring. A strong intra­molecular N—H⋯O hydrogen bond occurs in each mol­ecule. The crystal packing is stabilized by weak inter­molecular N—H⋯O and N—H⋯S hydrogen-bond inter­actions, the latter forming an infinite co-operative hydrogen-bonded two-dimensional network along [110].

## Related literature

For general background to the chemistry of thio­urea derivatives, see: Zhang *et al.* (2004 ▶); For related structures, see: Saeed *et al.* (2008*a* ▶,*b* ▶, 2009 ▶). For an ep­oxy resin curing agent, see: Saeed *et al.* (2009 ▶). For bond-length data, see: Allen *et al.* (1987 ▶).
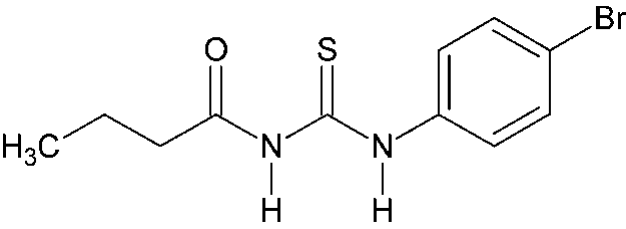


## Experimental

### 

#### Crystal data

C_11_H_13_BrN_2_OS
*M* *_r_* = 301.20
Triclinic, 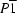

*a* = 6.1746 (3) Å
*b* = 10.7883 (4) Å
*c* = 19.6450 (8) Å
α = 87.719 (3)°
β = 81.557 (4)°
γ = 76.047 (4)°
*V* = 1256.23 (9) Å^3^
*Z* = 4
Mo *K*α radiation
μ = 3.42 mm^−1^
*T* = 123 K
0.53 × 0.24 × 0.11 mm

#### Data collection

Oxford Diffraction Xcalibur Ruby Gemini diffractometer
Absorption correction: multi-scan (*CrysAlis RED*; Oxford Diffraction, 2007 ▶) *T*_min_ = 0.187, *T*_max_ = 1.000
13276 measured reflections
5362 independent reflections
3535 reflections with *I* > 2σ(*I*)
*R*_int_ = 0.054

#### Refinement

*R*[*F* ^2^ > 2σ(*F* ^2^)] = 0.043
*wR*(*F* ^2^) = 0.095
*S* = 0.92
5362 reflections
307 parameters
18 restraints
H-atom parameters constrained
Δρ_max_ = 0.83 e Å^−3^
Δρ_min_ = −0.74 e Å^−3^

### 

Data collection: *CrysAlis PRO* (Oxford Diffraction, 2007 ▶); cell refinement: *CrysAlis RED* (Oxford Diffraction, 2007 ▶); data reduction: *CrysAlis RED*; program(s) used to solve structure: *SHELXS97* (Sheldrick, 2008 ▶); program(s) used to refine structure: *SHELXL97* (Sheldrick, 2008 ▶); molecular graphics: *SHELXTL* (Sheldrick, 2008 ▶); software used to prepare material for publication: *PLATON* (Spek, 2009 ▶).

## Supplementary Material

Crystal structure: contains datablocks global, I. DOI: 10.1107/S1600536810050373/im2247sup1.cif

Structure factors: contains datablocks I. DOI: 10.1107/S1600536810050373/im2247Isup2.hkl

Additional supplementary materials:  crystallographic information; 3D view; checkCIF report

## Figures and Tables

**Table 1:** Hydrogen-bond geometry (Å, °)

*D*—H⋯*A*	*D*—H	H⋯*A*	*D*⋯*A*	*D*—H⋯*A*
N1*A*—H1*AA*⋯O1*A*	0.88	1.97	2.666 (5)	135
N1*A*—H1*AA*⋯O1*A*^i^	0.88	2.36	3.083 (6)	140
N2*A*—H2*AB*⋯S1*A*^ii^	0.88	2.54	3.382 (4)	160
N1*B*—H1*BA*⋯O1*B*	0.88	1.98	2.662 (4)	134
N2*B*—H2*BB*⋯S1*B*^iii^	0.88	2.50	3.370 (3)	169

Symmetry codes: (i) 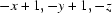; (ii) 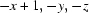; (iii) 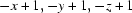.

**Fig. 1.**
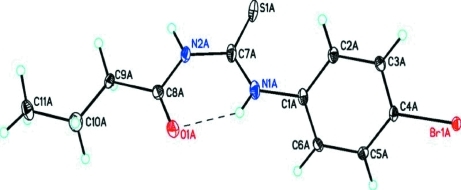
Molecular structure of C11H13BrN2OS, (A) showing the atom labeling scheme and 50% probability displacement ellipsoids. Dashed lined indicate intramolecular N—H···O hydrogen bonding. Only the predominate butanamide component (0.532 (6) occupancy) is displayed.

**Fig. 2.**
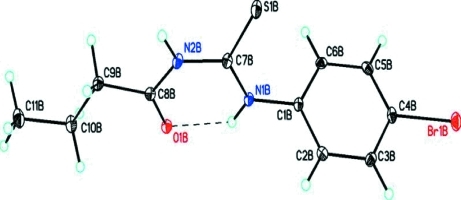
Molecular structure of C11H13BrN2OS, (B) showing the atom labeling scheme and 50% probability displacement ellipsoids. Dashed lined indicate intramolecular N—H···O hydrogen bonding.

**Fig. 3.**
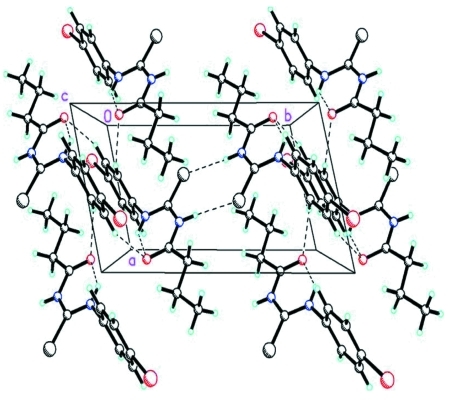
Packing diagram of the title compound viewed down the c axis. Dashed lines indicate strong N—H···O, weak N—H···O and N—H···S hydrogen bonds and are also displaying an R22(8) graph set motif betwen adjacent A–B molecules.

## supplementary crystallographic information

### Comment

The background to this study has been set in our previous work on the structural chemistry of N, N'-disubstituted thiourea (Saeed *et al.*, 2008*a*,*b*). Herein, as a continuation of these studies, the structure of the title compound, (I), C_11_H_13_BrN_2_OS, is described. With two molecules in the asymmetric unit, the dihedral angle between the mean planes of the benzene ring and carbamothioyl group is 63.66° (A) Fig. 1) and 80.3 (0)° (B) (Fig. 2), respectively. The butanamide group in A is disordered (0.532 (6) & 0.4686 occupancy). The carbamothioyl group is twisted by 63.6 (6)° (A) and 80.3 (0)° (B) from the mean plane of the respective benzene ring. Bond distances and angles are in normal ranges (Allen *et al.* , 1987). Crystal packing is stabilized by strong intramolecular N—H···O and weak intermolecular N—H···O and N—H···S hydrogen bond interactions, the latter forming an infinite cooperative hydrogen bonded 2-D network along 110. (Fig. 3).

### Experimental

A solution of butanoyl chloride (0.01 mol) in anhydrous acetone (75 ml) and 3% tetrabutylammonium bromide (TBAB) as a phase-transfer catalyst (PTC) in anhydrous acetone was added dropwise to a suspension of dry potassium thiocyanate (0.01 mol) in acetone (50 ml) and the reaction mixture was refluxed for 50 min. After cooling to room temperature, a solution of *p*-bromoaniline (0.01 mol) in anhydrous acetone (25 ml) was added dropwise and the resulting mixture refluxed for 3 h. Hydrochloric acid (0.1 N, 300 ml) was added, and the solution was filtered. The solid product was washed with water and purified by re-crystallization from ethyl acetate (yield: 92%).

### Refinement

N-H bond lengths were set to 0.88Å. All other H atoms were placed in calculated positions and then refined using the riding model approximation with atom–H lengths of 0.95 Å (CH), 0.99 Å (CH_2_), or 0.98 Å (CH_3_). Isotropic displacement parameters for these atoms were set to 1.2 (CH, CH_2_, NH) or 1.50 (CH_3_) times *U*_eq_ of the parent atom.

### Crystal data

**Table:**

C_11_H_13_BrN_2_OS	*Z* = 4
*M**_r_* = 301.20	*F*(000) = 608
Triclinic, *P*1	*D*_x_ = 1.593 Mg m^−^^3^
Hall symbol: -P 1	Melting point: 409 K
*a* = 6.1746 (3) Å	Mo *K*α radiation, λ = 0.71073 Å
*b* = 10.7883 (4) Å	Cell parameters from 6019 reflections
*c* = 19.6450 (8) Å	θ = 5.1–28.4°
α = 87.719 (3)°	µ = 3.42 mm^−^^1^
β = 81.557 (4)°	*T* = 123 K
γ = 76.047 (4)°	Prism, colorless
*V* = 1256.23 (9) Å^3^	0.53 × 0.24 × 0.11 mm

### Data collection

**Table:**

Oxford Diffraction Xcalibur Ruby Gemini diffractometer	5362 independent reflections
Radiation source: Enhance (Mo) X-ray Source	3535 reflections with *I* > 2σ(*I*)
graphite	*R*_int_ = 0.054
Detector resolution: 10.5081 pixels mm^-1^	θ_max_ = 28.5°, θ_min_ = 5.1°
ω scans	*h* = −8→8
Absorption correction: multi-scan (*CrysAlis RED*; Oxford Diffraction, 2007)	*k* = −13→14
*T*_min_ = 0.187, *T*_max_ = 1.000	*l* = −24→26
13276 measured reflections	

### Refinement

**Table:**

Refinement on *F*^2^	Primary atom site location: structure-invariant direct methods
Least-squares matrix: full	Secondary atom site location: difference Fourier map
*R*[*F*^2^ > 2σ(*F*^2^)] = 0.043	Hydrogen site location: inferred from neighbouring sites
*wR*(*F*^2^) = 0.095	H-atom parameters constrained
*S* = 0.92	*w* = 1/[σ^2^(*F*_o_^2^) + (0.0504*P*)^2^] where *P* = (*F*_o_^2^ + 2*F*_c_^2^)/3
5362 reflections	(Δ/σ)_max_ = 0.001
307 parameters	Δρ_max_ = 0.83 e Å^−^^3^
18 restraints	Δρ_min_ = −0.74 e Å^−^^3^

### Special details

**Table:**

Geometry. All e.s.d.'s (except the e.s.d. in the dihedral angle between two l.s. planes) are estimated using the full covariance matrix. The cell e.s.d.'s are taken into account individually in the estimation of e.s.d.'s in distances, angles and torsion angles; correlations between e.s.d.'s in cell parameters are only used when they are defined by crystal symmetry. An approximate (isotropic) treatment of cell e.s.d.'s is used for estimating e.s.d.'s involving l.s. planes.
Refinement. Refinement of *F*^2^ against ALL reflections. The weighted *R*-factor *wR* and goodness of fit *S* are based on *F*^2^, conventional *R*-factors *R* are based on *F*, with *F* set to zero for negative *F*^2^. The threshold expression of *F*^2^ > σ(*F*^2^) is used only for calculating *R*-factors(gt) *etc*. and is not relevant to the choice of reflections for refinement. *R*-factors based on *F*^2^ are statistically about twice as large as those based on *F*, and *R*- factors based on ALL data will be even larger.

### Fractional atomic coordinates and isotropic or equivalent isotropic displacement parameters (Å^2^)

**Table:**

	*x*	*y*	*z*	*U*_iso_*/*U*_eq_	Occ. (<1)
Br1A	1.12197 (6)	0.33692 (3)	0.30413 (2)	0.02775 (12)	
S1A	0.6977 (2)	0.04500 (13)	0.07060 (6)	0.0486 (3)	
O1A	0.3799 (6)	0.4183 (4)	−0.03038 (17)	0.0751 (13)	
N1A	0.6368 (5)	0.2974 (4)	0.06119 (16)	0.0360 (8)	
H1AA	0.5835	0.3690	0.0398	0.043*	
N2A	0.4761 (6)	0.2029 (4)	−0.01539 (17)	0.0454 (10)	0.468 (6)
H2AB	0.4692	0.1311	−0.0337	0.054*	0.468 (6)
N2C	0.4761 (6)	0.2029 (4)	−0.01539 (17)	0.0454 (10)	0.532 (6)
H2CA	0.4577	0.1312	−0.0308	0.054*	0.532 (6)
C1A	0.7542 (6)	0.3041 (4)	0.11839 (19)	0.0267 (9)	
C2A	0.9809 (6)	0.2465 (4)	0.11540 (19)	0.0276 (9)	
H2AA	1.0608	0.1997	0.0758	0.033*	
C3A	1.0908 (6)	0.2578 (4)	0.17075 (19)	0.0256 (9)	
H3AA	1.2470	0.2195	0.1694	0.031*	
C4A	0.9692 (6)	0.3256 (3)	0.22803 (19)	0.0239 (8)	
C5A	0.7432 (6)	0.3813 (3)	0.23116 (19)	0.0252 (9)	
H5AA	0.6621	0.4271	0.2710	0.030*	
C6A	0.6355 (6)	0.3701 (3)	0.1759 (2)	0.0264 (9)	
H6AA	0.4791	0.4080	0.1775	0.032*	
C7A	0.6030 (6)	0.1894 (5)	0.0382 (2)	0.0380 (11)	
C8A	0.359 (8)	0.3127 (6)	−0.044 (2)	0.066 (3)	0.468 (6)
C9A	0.1864 (14)	0.2620 (8)	−0.0861 (4)	0.0248 (13)	0.468 (6)
H9AA	0.2727	0.2012	−0.1225	0.030*	0.468 (6)
H9AB	0.0892	0.2179	−0.0543	0.030*	0.468 (6)
C10A	0.047 (4)	0.375 (3)	−0.1171 (9)	0.045 (3)	0.468 (6)
H10A	0.1473	0.4270	−0.1415	0.054*	0.468 (6)
H10B	−0.0541	0.4286	−0.0799	0.054*	0.468 (6)
C11A	−0.095 (6)	0.340 (4)	−0.1676 (15)	0.044 (5)	0.468 (6)
H11A	−0.1511	0.4151	−0.1954	0.066*	0.468 (6)
H11B	−0.2221	0.3113	−0.1419	0.066*	0.468 (6)
H11C	−0.0014	0.2714	−0.1977	0.066*	0.468 (6)
C8C	0.375 (7)	0.3120 (5)	−0.0481 (19)	0.066 (3)	0.532 (6)
C9C	0.2633 (12)	0.3155 (7)	−0.1089 (4)	0.0248 (13)	0.532 (6)
H9CA	0.2977	0.2288	−0.1287	0.030*	0.532 (6)
H9CB	0.3200	0.3727	−0.1441	0.030*	0.532 (6)
C10C	0.008 (4)	0.364 (2)	−0.0892 (8)	0.045 (3)	0.532 (6)
H10C	−0.0407	0.3218	−0.0460	0.054*	0.532 (6)
H10D	−0.0280	0.4570	−0.0809	0.054*	0.532 (6)
C11C	−0.119 (5)	0.338 (3)	−0.1444 (11)	0.044 (5)	0.532 (6)
H11D	−0.2794	0.3790	−0.1325	0.066*	0.532 (6)
H11E	−0.1006	0.2454	−0.1484	0.066*	0.532 (6)
H11F	−0.0604	0.3718	−0.1885	0.066*	0.532 (6)
Br1B	0.29893 (6)	−0.08278 (4)	0.22800 (2)	0.03337 (13)	
S1B	0.40894 (16)	0.38143 (8)	0.43013 (5)	0.0282 (2)	
O1B	0.9939 (4)	0.1368 (2)	0.52752 (12)	0.0214 (5)	
N1B	0.7056 (5)	0.1586 (3)	0.43688 (15)	0.0199 (7)	
H1BA	0.8214	0.1106	0.4543	0.024*	
N2B	0.7405 (5)	0.3202 (3)	0.50548 (14)	0.0214 (7)	
H2BB	0.6938	0.4015	0.5166	0.026*	
C1B	0.6082 (5)	0.1031 (3)	0.38714 (17)	0.0170 (8)	
C2B	0.7103 (6)	0.0919 (3)	0.31965 (18)	0.0199 (8)	
H2BA	0.8430	0.1217	0.3062	0.024*	
C3B	0.6197 (6)	0.0373 (3)	0.27136 (19)	0.0223 (8)	
H3BA	0.6885	0.0297	0.2247	0.027*	
C4B	0.4275 (6)	−0.0060 (3)	0.29247 (19)	0.0214 (8)	
C5B	0.3250 (6)	0.0038 (3)	0.35972 (19)	0.0222 (8)	
H5BA	0.1928	−0.0266	0.3732	0.027*	
C6B	0.4171 (6)	0.0584 (3)	0.40740 (19)	0.0217 (8)	
H6BA	0.3489	0.0652	0.4541	0.026*	
C7B	0.6293 (6)	0.2788 (3)	0.45781 (17)	0.0196 (8)	
C8B	0.9147 (5)	0.2507 (3)	0.53771 (17)	0.0188 (8)	
C9B	0.9965 (6)	0.3278 (3)	0.58645 (19)	0.0227 (8)	
H9BA	0.8748	0.3570	0.6252	0.027*	
H9BB	1.0303	0.4045	0.5620	0.027*	
C10B	1.2057 (6)	0.2531 (3)	0.6150 (2)	0.0286 (9)	
H10E	1.3226	0.2162	0.5763	0.034*	
H10F	1.1680	0.1817	0.6437	0.034*	
C11B	1.2993 (7)	0.3366 (4)	0.6579 (2)	0.0366 (10)	
H11G	1.4383	0.2862	0.6732	0.055*	
H11H	1.1881	0.3682	0.6981	0.055*	
H11I	1.3319	0.4090	0.6301	0.055*	

### Atomic displacement parameters (Å^2^)

**Table:**

	*U*^11^	*U*^22^	*U*^33^	*U*^12^	*U*^13^	*U*^23^
Br1A	0.0310 (2)	0.0221 (2)	0.0329 (2)	−0.00409 (16)	−0.01694 (17)	−0.00039 (17)
S1A	0.0591 (8)	0.0681 (8)	0.0314 (6)	−0.0304 (7)	−0.0239 (5)	0.0060 (6)
O1A	0.079 (3)	0.080 (3)	0.047 (2)	0.038 (2)	−0.039 (2)	−0.024 (2)
N1A	0.0339 (19)	0.055 (2)	0.0200 (18)	−0.0100 (17)	−0.0101 (15)	0.0062 (17)
N2A	0.033 (2)	0.071 (3)	0.026 (2)	0.0062 (19)	−0.0118 (16)	−0.0179 (19)
N2C	0.033 (2)	0.071 (3)	0.026 (2)	0.0062 (19)	−0.0118 (16)	−0.0179 (19)
C1A	0.030 (2)	0.035 (2)	0.017 (2)	−0.0117 (18)	−0.0078 (16)	0.0106 (17)
C2A	0.028 (2)	0.038 (2)	0.018 (2)	−0.0102 (18)	−0.0037 (16)	0.0027 (18)
C3A	0.0193 (19)	0.030 (2)	0.029 (2)	−0.0085 (17)	−0.0036 (16)	0.0036 (18)
C4A	0.030 (2)	0.0193 (19)	0.029 (2)	−0.0116 (17)	−0.0162 (17)	0.0082 (16)
C5A	0.027 (2)	0.0217 (19)	0.028 (2)	−0.0061 (17)	−0.0066 (17)	−0.0009 (16)
C6A	0.021 (2)	0.025 (2)	0.034 (2)	−0.0037 (16)	−0.0096 (17)	0.0024 (18)
C7A	0.024 (2)	0.070 (3)	0.020 (2)	−0.010 (2)	−0.0043 (17)	−0.003 (2)
C8A	0.050 (5)	0.091 (4)	0.038 (4)	0.037 (3)	−0.022 (4)	−0.034 (3)
C9A	0.032 (4)	0.025 (3)	0.022 (3)	−0.014 (2)	−0.007 (3)	−0.002 (3)
C10A	0.033 (8)	0.056 (6)	0.049 (11)	−0.013 (4)	−0.010 (8)	−0.004 (10)
C11A	0.036 (6)	0.049 (3)	0.055 (15)	−0.014 (4)	−0.024 (9)	0.009 (10)
C8C	0.050 (5)	0.091 (4)	0.038 (4)	0.037 (3)	−0.022 (4)	−0.034 (3)
C9C	0.032 (4)	0.025 (3)	0.022 (3)	−0.014 (2)	−0.007 (3)	−0.002 (3)
C10C	0.033 (8)	0.056 (6)	0.049 (11)	−0.013 (4)	−0.010 (8)	−0.004 (10)
C11C	0.036 (6)	0.049 (3)	0.055 (15)	−0.014 (4)	−0.024 (9)	0.009 (10)
Br1B	0.0354 (2)	0.0344 (2)	0.0352 (3)	−0.01105 (19)	−0.01454 (18)	−0.00672 (19)
S1B	0.0362 (6)	0.0152 (5)	0.0327 (6)	0.0040 (4)	−0.0195 (4)	−0.0041 (4)
O1B	0.0235 (13)	0.0133 (12)	0.0262 (14)	−0.0003 (11)	−0.0065 (11)	−0.0011 (11)
N1B	0.0211 (15)	0.0125 (15)	0.0247 (17)	0.0024 (12)	−0.0080 (13)	−0.0045 (13)
N2B	0.0286 (17)	0.0113 (14)	0.0233 (17)	0.0020 (13)	−0.0105 (13)	−0.0025 (13)
C1B	0.0212 (18)	0.0084 (16)	0.021 (2)	0.0001 (14)	−0.0076 (15)	0.0001 (14)
C2B	0.0202 (18)	0.0138 (17)	0.026 (2)	−0.0039 (15)	−0.0037 (15)	0.0014 (15)
C3B	0.026 (2)	0.0197 (19)	0.0192 (19)	−0.0004 (16)	−0.0036 (15)	−0.0008 (15)
C4B	0.027 (2)	0.0111 (17)	0.028 (2)	−0.0042 (15)	−0.0118 (16)	−0.0013 (15)
C5B	0.0173 (18)	0.0152 (18)	0.033 (2)	−0.0018 (15)	−0.0053 (16)	0.0019 (16)
C6B	0.0193 (19)	0.0188 (18)	0.023 (2)	0.0026 (15)	−0.0012 (15)	−0.0010 (16)
C7B	0.026 (2)	0.0151 (18)	0.0185 (19)	−0.0039 (16)	−0.0065 (16)	0.0019 (15)
C8B	0.0199 (18)	0.0179 (18)	0.0180 (19)	−0.0033 (16)	−0.0037 (15)	0.0029 (15)
C9B	0.0237 (19)	0.0149 (18)	0.028 (2)	0.0022 (15)	−0.0087 (16)	−0.0027 (15)
C10B	0.030 (2)	0.0149 (19)	0.041 (2)	0.0011 (16)	−0.0171 (18)	−0.0005 (17)
C11B	0.031 (2)	0.023 (2)	0.058 (3)	−0.0008 (18)	−0.022 (2)	−0.005 (2)

### Geometric parameters (Å, °)

**Table:**

Br1A—C4A	1.904 (3)	C10C—H10C	0.9900
S1A—C7A	1.663 (5)	C10C—H10D	0.9900
O1A—C8C	1.220 (4)	C11C—H11D	0.9800
O1A—C8A	1.220 (4)	C11C—H11E	0.9800
N1A—C7A	1.338 (5)	C11C—H11F	0.9800
N1A—C1A	1.437 (4)	Br1B—C4B	1.902 (3)
N1A—H1AA	0.8800	S1B—C7B	1.678 (3)
N2A—C8A	1.376 (4)	O1B—C8B	1.221 (3)
N2A—C7A	1.385 (5)	N1B—C7B	1.328 (4)
N2A—H2AB	0.8800	N1B—C1B	1.438 (4)
C1A—C6A	1.374 (5)	N1B—H1BA	0.8800
C1A—C2A	1.382 (5)	N2B—C8B	1.376 (4)
C2A—C3A	1.387 (5)	N2B—C7B	1.386 (4)
C2A—H2AA	0.9500	N2B—H2BB	0.8800
C3A—C4A	1.385 (5)	C1B—C2B	1.378 (5)
C3A—H3AA	0.9500	C1B—C6B	1.380 (5)
C4A—C5A	1.373 (5)	C2B—C3B	1.385 (5)
C5A—C6A	1.377 (5)	C2B—H2BA	0.9500
C5A—H5AA	0.9500	C3B—C4B	1.381 (5)
C6A—H6AA	0.9500	C3B—H3BA	0.9500
C8A—C9A	1.644 (14)	C4B—C5B	1.375 (5)
C9A—C10A	1.48 (3)	C5B—C6B	1.382 (5)
C9A—H9AA	0.9900	C5B—H5BA	0.9500
C9A—H9AB	0.9900	C6B—H6BA	0.9500
C10A—C11A	1.53 (5)	C8B—C9B	1.508 (4)
C10A—H10A	0.9900	C9B—C10B	1.518 (5)
C10A—H10B	0.9900	C9B—H9BA	0.9900
C11A—H11A	0.9800	C9B—H9BB	0.9900
C11A—H11B	0.9800	C10B—C11B	1.521 (5)
C11A—H11C	0.9800	C10B—H10E	0.9900
C8C—C9C	1.459 (11)	C10B—H10F	0.9900
C9C—C10C	1.53 (2)	C11B—H11G	0.9800
C9C—H9CA	0.9900	C11B—H11H	0.9800
C9C—H9CB	0.9900	C11B—H11I	0.9800
C10C—C11C	1.50 (4)		
			
C8C—O1A—C8A	5(6)	H10C—C10C—H10D	107.9
C7A—N1A—C1A	124.4 (4)	C10C—C11C—H11D	109.5
C7A—N1A—H1AA	117.8	C10C—C11C—H11E	109.5
C1A—N1A—H1AA	117.8	H11D—C11C—H11E	109.5
C8A—N2A—C7A	129.2 (5)	C10C—C11C—H11F	109.5
C8A—N2A—H2AB	115.4	H11D—C11C—H11F	109.5
C7A—N2A—H2AB	115.4	H11E—C11C—H11F	109.5
C6A—C1A—C2A	120.9 (3)	C7B—N1B—C1B	123.1 (3)
C6A—C1A—N1A	118.3 (3)	C7B—N1B—H1BA	118.4
C2A—C1A—N1A	120.8 (3)	C1B—N1B—H1BA	118.4
C1A—C2A—C3A	119.4 (3)	C8B—N2B—C7B	128.4 (3)
C1A—C2A—H2AA	120.3	C8B—N2B—H2BB	115.8
C3A—C2A—H2AA	120.3	C7B—N2B—H2BB	115.8
C4A—C3A—C2A	118.9 (3)	C2B—C1B—C6B	120.4 (3)
C4A—C3A—H3AA	120.5	C2B—C1B—N1B	119.6 (3)
C2A—C3A—H3AA	120.5	C6B—C1B—N1B	120.0 (3)
C5A—C4A—C3A	121.5 (3)	C1B—C2B—C3B	120.2 (3)
C5A—C4A—Br1A	120.3 (3)	C1B—C2B—H2BA	119.9
C3A—C4A—Br1A	118.2 (3)	C3B—C2B—H2BA	119.9
C4A—C5A—C6A	119.2 (3)	C4B—C3B—C2B	118.6 (3)
C4A—C5A—H5AA	120.4	C4B—C3B—H3BA	120.7
C6A—C5A—H5AA	120.4	C2B—C3B—H3BA	120.7
C1A—C6A—C5A	120.0 (3)	C5B—C4B—C3B	121.8 (3)
C1A—C6A—H6AA	120.0	C5B—C4B—Br1B	118.3 (3)
C5A—C6A—H6AA	120.0	C3B—C4B—Br1B	120.0 (3)
N1A—C7A—N2A	116.0 (4)	C4B—C5B—C6B	119.0 (3)
N1A—C7A—S1A	124.5 (3)	C4B—C5B—H5BA	120.5
N2A—C7A—S1A	119.5 (3)	C6B—C5B—H5BA	120.5
O1A—C8A—N2A	122.2 (5)	C1B—C6B—C5B	120.0 (3)
O1A—C8A—C9A	133.9 (10)	C1B—C6B—H6BA	120.0
N2A—C8A—C9A	103.4 (6)	C5B—C6B—H6BA	120.0
C10A—C9A—C8A	107.3 (12)	N1B—C7B—N2B	117.1 (3)
C10A—C9A—H9AA	110.3	N1B—C7B—S1B	124.1 (3)
C8A—C9A—H9AA	110.3	N2B—C7B—S1B	118.9 (2)
C10A—C9A—H9AB	110.3	O1B—C8B—N2B	122.5 (3)
C8A—C9A—H9AB	110.3	O1B—C8B—C9B	123.6 (3)
H9AA—C9A—H9AB	108.5	N2B—C8B—C9B	113.9 (3)
C9A—C10A—C11A	113 (2)	C8B—C9B—C10B	112.9 (3)
C9A—C10A—H10A	109.0	C8B—C9B—H9BA	109.0
C11A—C10A—H10A	109.0	C10B—C9B—H9BA	109.0
C9A—C10A—H10B	109.0	C8B—C9B—H9BB	109.0
C11A—C10A—H10B	109.0	C10B—C9B—H9BB	109.0
H10A—C10A—H10B	107.8	H9BA—C9B—H9BB	107.8
O1A—C8C—C9C	112.4 (6)	C9B—C10B—C11B	111.9 (3)
C8C—C9C—C10C	110 (2)	C9B—C10B—H10E	109.2
C8C—C9C—H9CA	109.7	C11B—C10B—H10E	109.2
C10C—C9C—H9CA	109.7	C9B—C10B—H10F	109.2
C8C—C9C—H9CB	109.7	C11B—C10B—H10F	109.2
C10C—C9C—H9CB	109.7	H10E—C10B—H10F	107.9
H9CA—C9C—H9CB	108.2	C10B—C11B—H11G	109.5
C11C—C10C—C9C	111.9 (16)	C10B—C11B—H11H	109.5
C11C—C10C—H10C	109.2	H11G—C11B—H11H	109.5
C9C—C10C—H10C	109.2	C10B—C11B—H11I	109.5
C11C—C10C—H10D	109.2	H11G—C11B—H11I	109.5
C9C—C10C—H10D	109.2	H11H—C11B—H11I	109.5
			
C7A—N1A—C1A—C6A	−115.5 (4)	O1A—C8C—C9C—C10C	−74 (4)
C7A—N1A—C1A—C2A	65.0 (5)	C8C—C9C—C10C—C11C	−165.1 (18)
C6A—C1A—C2A—C3A	−1.3 (6)	C7B—N1B—C1B—C2B	100.1 (4)
N1A—C1A—C2A—C3A	178.2 (3)	C7B—N1B—C1B—C6B	−81.5 (4)
C1A—C2A—C3A—C4A	0.7 (5)	C6B—C1B—C2B—C3B	0.9 (5)
C2A—C3A—C4A—C5A	0.2 (5)	N1B—C1B—C2B—C3B	179.2 (3)
C2A—C3A—C4A—Br1A	178.8 (3)	C1B—C2B—C3B—C4B	−0.4 (5)
C3A—C4A—C5A—C6A	−0.4 (6)	C2B—C3B—C4B—C5B	0.0 (5)
Br1A—C4A—C5A—C6A	−179.0 (3)	C2B—C3B—C4B—Br1B	−179.5 (2)
C2A—C1A—C6A—C5A	1.1 (6)	C3B—C4B—C5B—C6B	0.0 (5)
N1A—C1A—C6A—C5A	−178.5 (3)	Br1B—C4B—C5B—C6B	179.5 (2)
C4A—C5A—C6A—C1A	−0.2 (5)	C2B—C1B—C6B—C5B	−1.0 (5)
C1A—N1A—C7A—N2A	176.9 (3)	N1B—C1B—C6B—C5B	−179.3 (3)
C1A—N1A—C7A—S1A	−1.9 (5)	C4B—C5B—C6B—C1B	0.5 (5)
C8A—N2A—C7A—N1A	−8(3)	C1B—N1B—C7B—N2B	−179.4 (3)
C8A—N2A—C7A—S1A	171 (3)	C1B—N1B—C7B—S1B	0.7 (5)
C8C—O1A—C8A—N2A	85 (4)	C8B—N2B—C7B—N1B	−4.1 (5)
C8C—O1A—C8A—C9A	−104 (7)	C8B—N2B—C7B—S1B	175.8 (3)
C7A—N2A—C8A—O1A	11 (7)	C7B—N2B—C8B—O1B	−0.8 (6)
C7A—N2A—C8A—C9A	−162.5 (8)	C7B—N2B—C8B—C9B	179.5 (3)
O1A—C8A—C9A—C10A	6(7)	O1B—C8B—C9B—C10B	6.9 (5)
N2A—C8A—C9A—C10A	178 (3)	N2B—C8B—C9B—C10B	−173.3 (3)
C8A—C9A—C10A—C11A	171 (3)	C8B—C9B—C10B—C11B	174.0 (3)
C8A—O1A—C8C—C9C	98 (3)		

### Hydrogen-bond geometry (Å, °)

**Table:**

*D*—H···*A*	*D*—H	H···*A*	*D*···*A*	*D*—H···*A*
N1A—H1AA···O1A	0.88	1.97	2.666 (5)	135.
N1A—H1AA···O1A^i^	0.88	2.36	3.083 (6)	140.
N2A—H2AB···S1A^ii^	0.88	2.54	3.382 (4)	160.
N1B—H1BA···O1B	0.88	1.98	2.662 (4)	134.
N2B—H2BB···S1B^iii^	0.88	2.50	3.370 (3)	169.

Symmetry codes: (i) −*x*+1, −*y*+1, −*z*; (ii) −*x*+1, −*y*, −*z*; (iii) −*x*+1, −*y*+1, −*z*+1.
